# The altered multiscale dynamics of spontaneous brain activity in depression with Parkinson’s disease

**DOI:** 10.1007/s10072-022-05974-4

**Published:** 2022-03-02

**Authors:** Zhu Liu, Dongning Su, Lingyan Ma, Huimin Chen, Jinping Fang, Huizi Ma, Junhong Zhou, Tao Feng

**Affiliations:** 1 Department of Neurology, Movement Disorder Department, Fengtai District, Neurology Center, Capital Medical University, Beijing Tiantan Hospital, No.119, South 4th Ring West Road, Beijing, People’s Republic of China; 2 China National Clinical Research Center for Neurological Diseases, Beijing, China; 3 Hinda and Arthur Marcus Institute for Aging Research, Hebrew SeniorLife, 1200 Centre Street, Roslindale, Boston, MA USA; 4 Harvard Medical School, Boston, MA USA; 5 Beijing Rehabilitation Hospital of Capital Medical University, Beijing, China

**Keywords:** Depression in Parkinson’s disease, Resting-state BOLD fluctuation, Complexity, Multiscale entropy, Global functional connectivity

## Abstract

**Background:**

Depression is one typical mood disorder in Parkinson’s disease (DPD). The alterations in the resting-state brain activities are believed to be associated with DPD. These resting-state activities are regulated by neurophysiological components over multiple temporal scales. The multiscale dynamics of these spontaneous fluctuations are thus complex, but not well-characterized.

**Objective:**

To characterize the complexity of the spontaneous blood-oxygen-level-dependent (BOLD) of fMRI in DPD. We hypothesized that (1) compared to non-depression PD (NDPD), the complexity in DPD would be lower; and (2) the diminished complexity would be associated with lower connections/communications between brain regions.

**Methods:**

Twenty-nine participants (10 in DPD and 19 in NDPD) who were naïve to medications completed a resting-sate functional MRI scan. The BOLD complexity within each voxel was calculated by using multiscale entropy (MSE). The complexity of the whole brain and each of the 90 regions parcellated following automated-anatomical-labeling template was then obtained by averaging voxel-wised complexity across all brain regions or within each region. The level of connections of regions with diminished complexity was measured by their own global functional connectivity (FC).

**Results:**

As compared to NDPD patients, the whole-brain complexity and complexity in 18 regions were significantly lower in DPD (*F* > 16.3, *p* < 0.0005). Particularly, in eight of the 18 regions, lower complexity was associated with lower global FC (Beta = 0.333 ~ 0.611, *p* = 0.000 ~ 0.030).

**Conclusion:**

The results from this pilot study suggest that the resting-state BOLD complexity may provide critical knowledge into the pathology of DPD. Future studies are thus warranted to confirm the findings of this study.

**Supplementary Information:**

The online version contains supplementary material available at 10.1007/s10072-022-05974-4.

## Introduction

Parkinson’s disease (PD), in addition to manifesting with the cardinal motor symptoms (tremor, rigidity, bradykinesia) and other frequent motor features such as freezing of gait, is often accompanied with non-motor features including cognitive and affective disturbances. More than 30% of people with PD suffer from depression [1, 2], and mounting evidence has showed that the depression in PD (DPD) is oftentimes accompanied with apathy and anhedonia [3], and associated with elevated risk of dementia, more severe motor dysfunction and diminished quality of life in this population [4, 5]. It is thus important to better understand the underlying neurophysiological mechanisms of DPD, which can ultimately help the management of DPD and optimize the design of therapeutic strategies for DPD.

Depression is a mood disorder that is linked to abnormal regulation within the cortical networks of the brain. Evidence suggests that compared to those in non-depression PD (NDPD), people in DPD would loss dopamine transporter availability in the striatum and limbic brain regions, but not the serotonergic systems, of which the changes are oftentimes observed in people with depression only [3, 6]. Many neuroimaging studies have characterized the resting-state brain activities in DPD. By characterizing the amplitude of low-frequency fluctuation (ALFF) of functional MRI (fMRI) resting-state blood-oxygen-level-dependent (BOLD) signal, for example, it was observed that as compared to non-depression PD (NDPD) cohort, those with DPD had higher ALFF in the left orbitofrontal [7] and right subgenual cingulate cortex [8]. In another study, Luo and colleagues characterized the functional connectivity (FC) between cortical networks and observed that in DPD, the FC within the prefrontal-limbic network is reduced as compared to NDPD [7].

However, these techniques are based upon a single-scale measure (e.g., ALFF) or focus on the relationship between networks (e.g., FC); the interaction and communication between neurons or functional networks at resting state are over multiple scales of time, ranging from millisecond (e.g., the time to transmit neural impulses) to hours or days (e.g., circadian rhythms). For example, multiple neural circuits are associated with the regulation of mood, including the frontal-subcortical, basal ganglic-thalamic-cortical circuit [9–12]. The homeostatic mechanisms underlying neuronal/synaptic activity in depression range from minutes (e.g., dynamic enhancement/reduction of the postsynaptic response) to hours (e.g., strengthening/weakening of synaptic connections and neurochemical alterations including synaptic insertion and removal of neurotransmitters’ receptors) [13]. The complex *multiscale dynamics* of resting-state BOLD fluctuation thus contain critical information to the neuro-physiological procedures pertaining to the regulation of mental and physical functions. Several studies have measured the complexity of the multiscale dynamics in resting-state BOLD fluctuations using well-established non-linear techniques, such as multiscale entropy (MSE), and have linked such complexity to important functions [14–17]. Zhou et al., for example, observed that in older adults, lower complexity of the brain functional networks (e.g., motor and attention networks) was associated with slower walking speeds [14]. However, the complexity of resting-state BOLD fluctuations in DPD is still not well-characterized.

In this study, we primarily aimed to characterize the complexity of resting-state BOLD fluctuation in DPD, to identify the regions with significantly lower BOLD complexity in DPD, and to examine the potential factors that are pertaining to decreased complexity in DPD. Specifically, we hypothesize that (1) compared to those with NDPD, the whole-brain BOLD complexity (i.e., complexity averaged across all the brain regions) would be lower in participants with DPD;(2) the complexity of regions pertaining to mood regulation (e.g., prefrontal lobe, anterior cingulate cortex) would be lower; and (3) lower BOLD complexity of the identified region would be associated with less connections/interactions between this region and other regions, which was measured by global FC (i.e., the functional connectivity of the region of interest (ROI) to all the other brain regions).

## Material and methods

### Participants

Participants were recruited via the inpatient Department of Neurology. Study team first searched the data depository of patients who had the newly diagnosed PD on their clinical visit following the Movement Disorder Society (MDS) Clinical Diagnostic Criteria [18]. Study team then reached out to those who expressed the interests to future studies. The inclusion criteria for both DPD and NDPD were (1) to prevent the potential influence of medication on resting-state brain activities; the diagnosis of PD was completed within 1 week in prior to the study, so *all the participants were “naïve” to the PD medications, with medication history less than one week*; (2) right-handed. The inclusion criteria for DPD cohort were (1) depression as characterized according to the Diagnostic and Statistical Manual of Mental Disorders, fifth edition (DSM-V) [4, 19], that is, depressed mood and/or loss of interest or pleasure in life activities for at least 2 weeks and at least five symptoms listed on DSM-V that may cause significant impairment in functioning, and (2) *naïve to anti-depression medication (i.e., never taking anti-depression medication)*. Those who were not characterized as depression were then in NDPD. The exclusion criteria for both groups were (1) uncertain diagnosis for PD or suspicious of parkinsonism syndrome (e.g., vascular, drug- and toxin-induced, post-infectious parkinsonism), multiple system atrophy, cortico-basal ganglionic degeneration, or progressive supranuclear palsy; (2) history of stroke, head trauma, hydrocephalus, brain surgery, or brain tumor; (3) other kind of psychiatric diseases (e.g., anxiety); (4) self-reported cardiovascular diseases that may be related to the cerebral alterations (e.g., uncontrolled hypertension, congestive heart failure); ( 5) impaired cognitive function (i.e., score of the Chinese version of Mini–Mental State Exam (MMSE) [20] ≤ 24); (6) contraindications to MRI (e.g., metallic or electrical bio-implants, claustrophobia); and (7) inability to understand the study protocol. All the participants provided the written informed consent following the Helsinki Declaration (the registration number of this study was KY2021-004–02).

### Study protocol

After screening, 30 participants (10 in DPD and 20 in NDPD) were eligible for this study. All the assessments were completed in *medication-off* state, that is, the assessments were completed around 7:30 am in the early morning before any medication was taken on the day and at least 12 h after the last use of medication. Specifically, each participant completed the part III of MDS-Unified Parkinson’s Disease Rating Scale (MDS-UPDRS-III) assessing the severity of PD, and a 24-item Hamilton Depression Scale (HAMD) for the level of depression. The course of PD was also determined according to the self-reported time when the movement disorders (e.g., tremor, bradykinesia) were presented in participants. Then they completed an MRI scan to measure the resting-state BOLD fluctuations.

### MRI scan

The MRI scans were completed using a SIEMENS Trio 3-Tesla scanner (Siemens, Erlangen, Germany) with 20-channel head coil. Participants were instructed to relax, keep eyes closed but not fall asleep, and move as little as possible during MRI acquisition. The structural brain MRI data were acquired using T1-weighted, sagittal 3D magnetization-prepared rapid-gradient echo (MPRAGE) sequences with the following parameters: repetition time (TR)/echo time (TE)/inversion time = 2000 ms/2.19 ms/900 ms, flip angle (FA) = 9°, field of view (FOV) = 224 mm × 256 mm, in-plane resolution = 224 × 256, slice thickness = 1 mm, and 176 sagittal slices. The fMRI data were axially collected using an echo-planar imaging (EPI) sequence with the following parameters: TR/TE = 2500 ms/30 ms, FA = 90°, FOV = 200 mm × 200 mm, resolution = 70 × 70, axial slices = 43, thickness = 3 mm, and bandwidth = 1786 Hz/pixel.

### Data analysis

#### Pre-processing of fMRI data

The acquired resting-state BOLD signals were pre-processed using Resting-State fMRI Data Analysis Toolkit V1.24 (RESTplus V1.24, http://www.restfmri.net/forum/sites/default/files/200902_1511_RESTplus_v1.24.tar.gz) [25]. The acquired data were first transformed into the NIFTI format, and the first 10 volumes of each BOLD signal were excluded for magnetization stabilization. The following steps were then performed: slice-time correction, motion correction, spatially normalization using the EPI MNI template, 8-mm kernel smoothing, and scaling to a percentage change from the mean.

For the analysis of multiscale entropy (MSE), the BOLD time-series were high-pass–filtered using cut-off frequency of 0.01 Hz and entered into a general linear model to remove the effects of 24°of motion and their derivatives, nuisance CSF, and white matter. For the analysis of global functional connectivity (global FC), the BOLD time-series were band-pass–filtered from 0.01 to 0.08 Hz and entered into a general linear model to remove the effects of 24° of motion and their derivatives, nuisance CSF, white matter, and global signal. The residual time series in each voxel from this deconvolve was then used to calculate the MSE and global FC.

#### BOLD complexity

The complexity was first quantified at each brain voxel by using MSE [9, 12, 13] to calculate the entropies across five temporal scales (see details of MSE in Supplementary materials). After obtaining the voxel-wised complexity, the *whole-brain complexity* was then obtained by averaging the complexity index across all the voxels. For the cluster-based analysis, we parcellated the brain into 90 regions using the automated anatomical labeling (AAL-90) AAL-90 template. This widely-used brain parcellation offered a predefined reliable and unbiased localization of “functional” clusters with a good anatomical interpretability to identify functional change in psychological disorders [21]. Then complexity of each AAL-90 cluster (i.e., *regional complexity*) was then obtained by averaging the complexity index within each region. The whole-brain and regional complexity were used in the following analyses.

#### Global functional connectivity

The analyses of functional connectivity (FC) were performed using REST plus toolbox [22]. After identifying the regions with significantly decreased complexity (i.e., regions of interests, ROI) in DPD, we calculated the FCs of the ROI to each of the other 89 clusters, respectively. The global FC of each ROI was then obtained by averaging those 89 FCs.

### Statistical analysis

All the statistical analyses were performed using SPSS 25.0. To examine the differences in the demographic and clinical characteristics between groups, two-way ANOVA models were used. The model factor was group (i.e., DPD and NDPD) and the dependent variable was each outcome (e.g., age, UPDRS-III score) in separate models. The effect size of the significant differences was assessed using Hedges’ g, in which the small, medium, and large effect size was characterized by Hedges’ g =  > 0.2, =  > 0.5, and =  > 0.8, respectively.

To examine difference in the BOLD complexity (i.e., whole-brain complexity and regional complexity) between DPD and NDPD groups, the general linear models were used. The covariates of these models were age, sex, and the course of PD disease. Secondarily, we also performed similar analyses on the global FC of ROIs. To account for the potential multiple-comparison bias, the significance level for the models to compare the regional complexity was set to *p* < 0.001.

To examine association between BOLD complexity in the identified regions and their global FC, linear regression models were used. We performed the regression analyses on the total population (i.e., across both DPD and NDPD) by adjusting for age, sex, course of PD, and group (i.e., NDPD and DPD), and also within each cohort (i.e., within NDPD or DPD) by adjusting for age, sex, and course of PD.

## Results

All the 30 participants completed this study. One participant in NDPD group was excluded in the analysis because of the poor imaging quality due to the excessive head motion during MRI scan. Table 1 showed the demographic and clinical information of the 29 participants (10 in DPD and 19 in NDPD group). ANOVA models showed no significant differences in age, UPDRS-III score, course of PD, and cognitive function (i.e., MMSE score) between the DPD and NDPD groups. As expected, the score of HAMD in DPD group was significantly higher as compared to the NDPD group (*F* = 67.1, *p* < 0.0001).

**Table 1:** Demographic and clinical characteristics of PD patients with and without depression

	DPD (*N* = 10)	NDPD (*N* = 19)	*P*
Age (years)	59.79 ± 10.45	63.40 ± 12.27	0.41
Sex(female/male)	6/4	8/11	0.36
Course of PD (years)	2.55 ± 2.98	2.15 ± 1.77	0.65
MDS-UPDRS III	21.75 ± 7.26	18.89 ± 7.07	0.32
MMSE	26.90 ± 2.33	27.84 ± 1.57	0.21
HAMD	12 (11, 26)	3.5 (2, 5)	< 0.0001*
Whole-brain complexity	1.2 ± 0.03	1.26 ± 0.03	0.0005*

*DPD*, Parkinson’s disease with depression; *NDPD*, Parkinson’s disease without depression; *MDS-UPDRS III*, part III of MDS-Unified Parkinson’s Disease Rating Scale (MDS-UPDRS); *MMSE*, Mini–Mental State Exam; *HAMD*, Hamilton Depression Scale.*p < 0.05

### The effects of depression on the complexity of resting-state BOLD fluctuations

The ANOVA model demonstrated that as compared to NPDP, the whole-brain complexity as obtained by averaging the complexity of voxels across all the brain regions in DPD was significantly lower (*F* = 16.3, *p* = 0.0005, Hedges’ g = 2.0, Table 1), and this significance was independent from age, sex, and the course of PD.

**Fig. 1:**
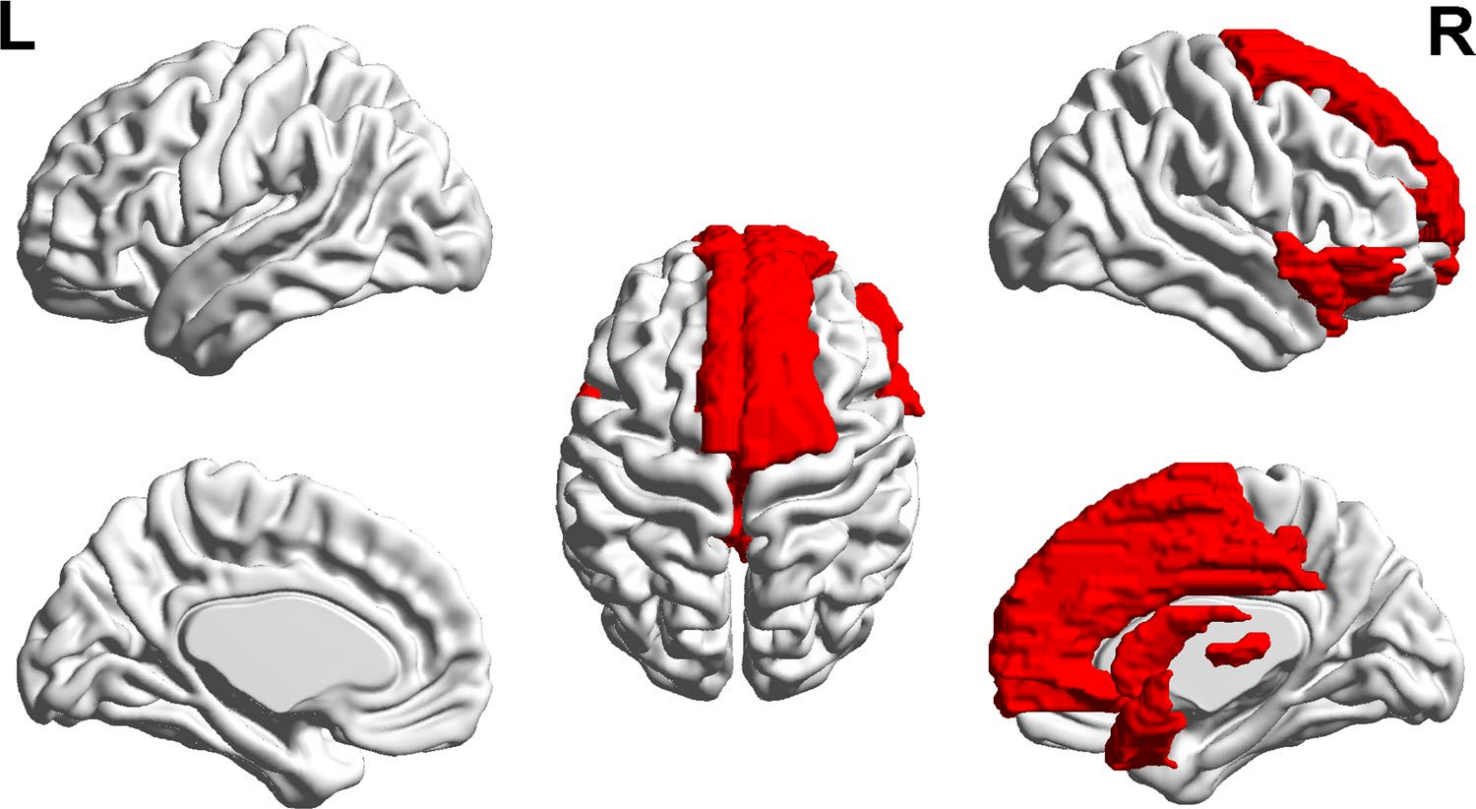
The statistical parametric map of clusters (in red) with significant difference in BOLD complexity between DPD and NDPD group

For the cluster-based analysis, the AAL template parcellated the brain into 90 clusters and the general linear models were used to compare the regional complexity between DPD and NDPD (Supplementary Table 1). It was observed that compared to the NDPD group, the complexity within 18 regions, which were pertaining to cognition and behavior, was significantly lower in DPD (Fig. 1, *p* < 0.001, Hedges’ g = 0.363–2.159). Specifically, these regions included right dorsolateral of superior frontal gyrus (SFG), right orbital part of inferior frontal gyrus (IFGorb), bilateral supplementary motor area (SMA), bilateral middle frontal gyrus (MFG), bilateral medial of superior frontal gyrus (SFGmed), right medial orbital of superior frontal gyrus (SFGmorb), bilateral anterior cingulate and paracingulate gyri (ACC), bilateral median cingulate and paracingulate gyri (MCC), right thalamus (THA), left heschl gyrus (HES), left superior temporal gyrus (STG), and bilateral temporal pole of superior temporal gyrus (STGp) (as highlighted in Fig. 1).

**Fig. 2:**
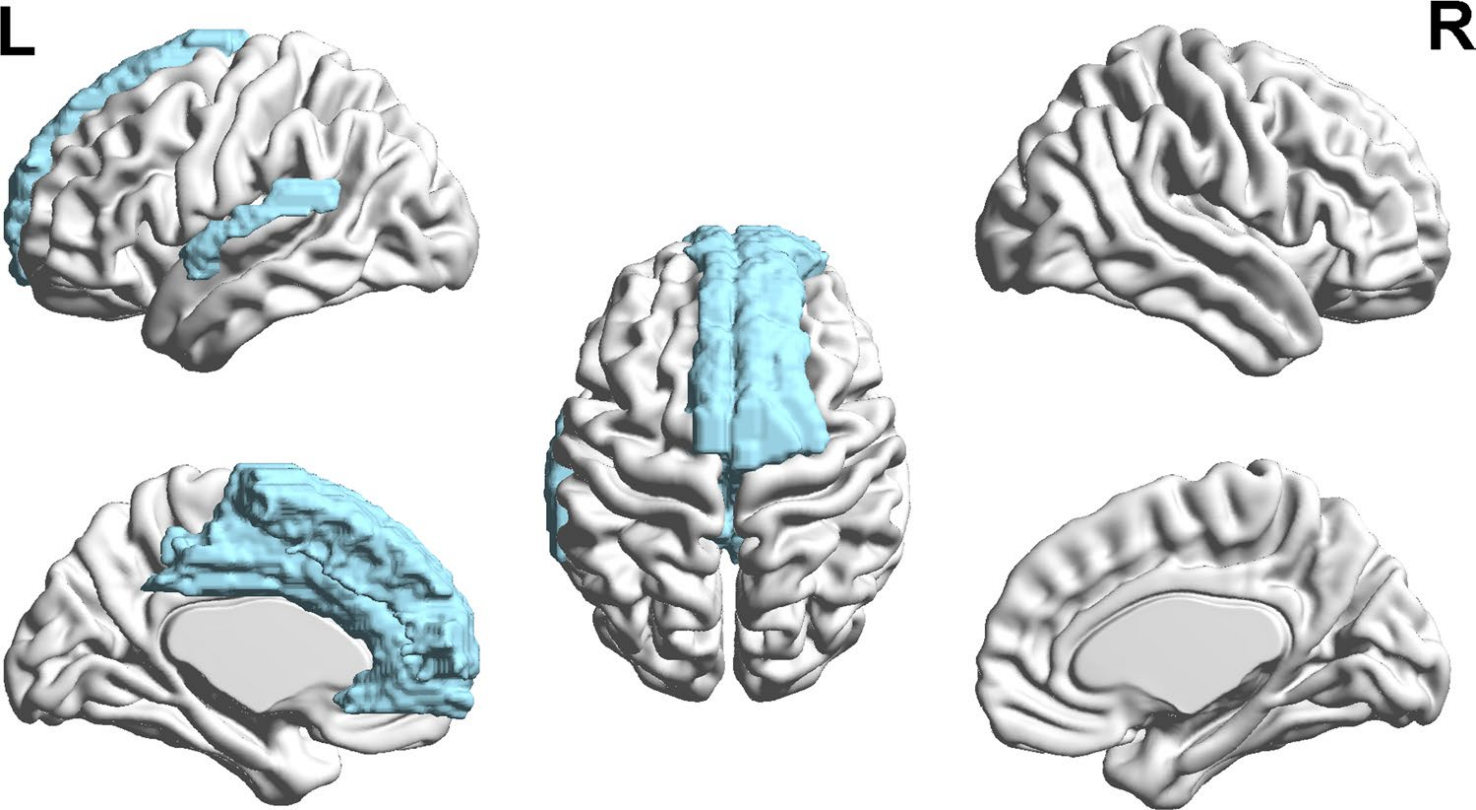
The statistical parametric map of clusters (in blue) with significant difference in global functional connectivity between DPD and NDPD group

Within these 18 identified regions, it was observed that compared to NDPD, the global FC of 13 of them was lower in DPD (Fig.2, *F* > 4.9, *p* < 0.04, Hedges’ g = 0.227–1.304), including right SFG, bilateral SMA, bilateral MFG, bilateral SFGmed, right SFGmorb, bilateral ACC, bilateral MCC, and left STG.

### The association between complexity and global functional connectivity

**Fig. 3:**
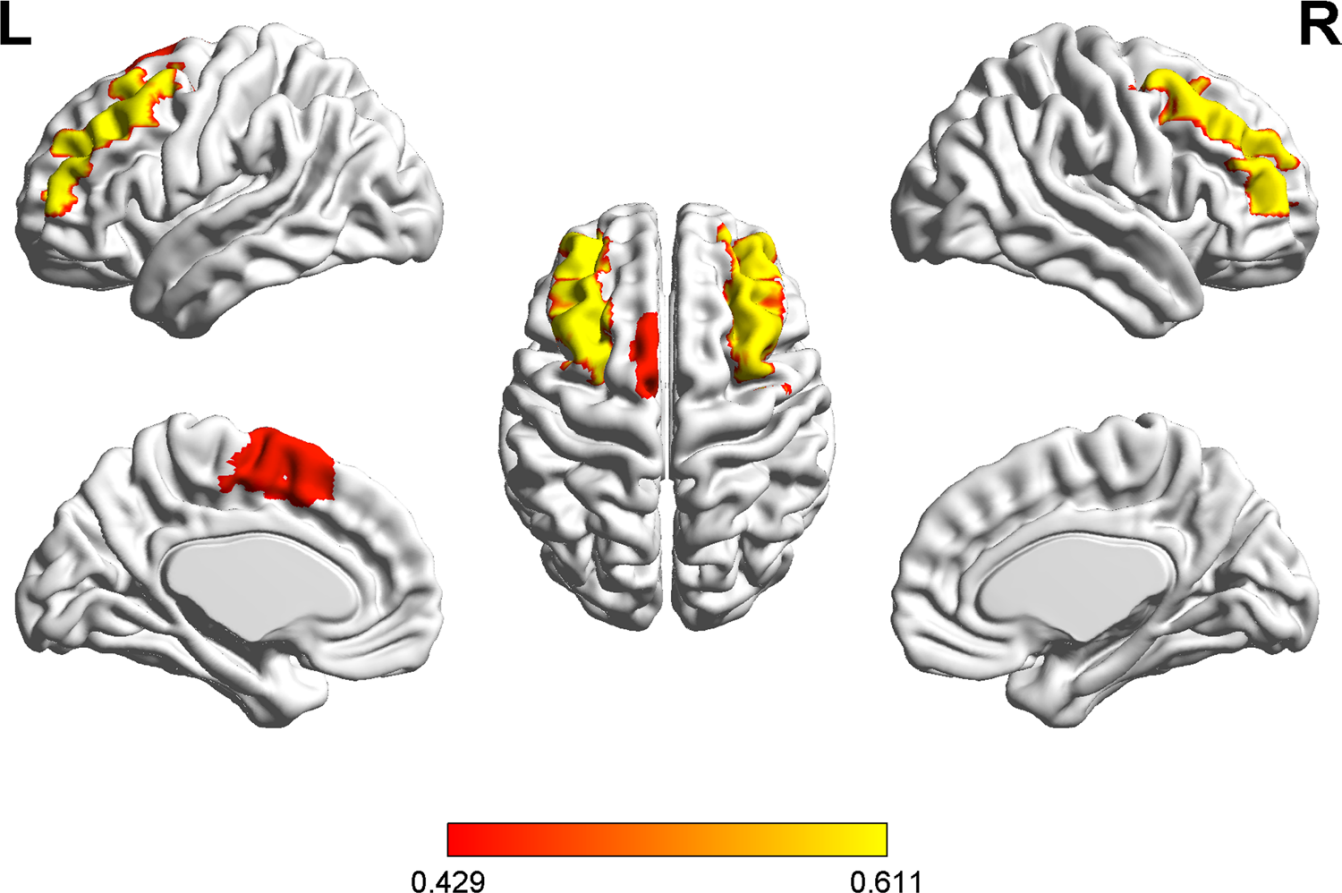
The statistical parametric map of the association between resting-state complexity and global FC

To explore the potential underlying mechanisms through which the BOLD complexity is diminished in DPD, the linear regression models (Table 2, Fig. 3) adjusted for age, sex, course of PD, and group demonstrated that the complexity in eight of the identified regions (i.e., right SFG, bilateral MFG, right IFGorb, left SMA, right SFGmed, bilateral MCC) was significantly associated with their global FC, respectively (standardized beta = 0.333 ~ 0.611, *p* = 0.000 ~ 0.030). The less the global FC within these regions, the lower the complexity.

**Table 2:** The association between resting-state complexity and global FC within each region with significantly lower complexity in DPD across all the participants

AAL-90	Area	Standardized beta	*T* value	*P* value
4	SFG.R	0.362	2.408	0.023^#^
7	MFG.L	0.611	4.011	0.000^*^
8	MFG.R	0.601	3.904	0.001^*^
16	IFGorb.R	0.390	2.681	0.013^#^
19	SMA.L	0.450	2.974	0.006^*^
20	SMA.R	0.219	1.468	0.154
23	SFGmed.L	0.247	1.651	0.111
24	SFGmed.R	0.333	2.295	0.030^#^
26	SFGmorb.R	0.200	1.236	0.228
31	ACC.L	0.222	1.457	0.157
32	ACC.R	0.237	1.840	0.770
33	MCC.L	0.429	2.996	0.006^*^
34	MCC.R	0.355	2.442	0.022^#^
78	THA.R	0.163	1.437	0.883
79	HES.L	0.164	1.101	0.281
81	STG.L	0.298	2.022	0.054
83	STGp.L	0.177	1.141	0.264
84	STGp.R	0.259	1.807	0.083

*AAL-90*, labels of the automated anatomical labeling atlas. *SFG*, superior frontal gyrus, dorsolateral. *MFG*, middle frontal gyrus. *IFGorb*, Inferior frontal gyrus, orbital part. *SMA*, supplementary motor area. *SFGmed*, superior frontal gyrus, medial. *SFGmorb*, superior frontal gyrus, medial orbital. *ACC*, anterior cingulate and paracingulate gyri. *MCC*, median cingulate and paracingulate gyri. *THA*, thalamus. *HES*, Heschl gyrus. *STG*, superior temporal gyrus. *STGp*, temporal pole, superior temporal gyrus. ^#^: *P* < 0.05, ^*^: *P* < 0.01

Secondarily, similar relationship was observed within DPD group. Those with less global FC within left SMA, bilateral SFGmed, bilateral MFG, left ACC, bilateral MCC, and left STGp had lower complexity within each of these regions (standardized beta = 0.434 ~ 0.771, *p* = 0.004 ~ 0.041), respectively. Within the NDPD group, it was observed in only left SMA that the lower complexity was significantly associated with less global FC (standardized beta = 0.528, *p* = 0.020).

## Discussion

To our knowledge, this pilot study is the first to characterize the multiscale dynamics of spontaneous brain activities in people with DPD, and to explore the underlying mechanism that may contribute to such altered dynamics in this cohort. It is observed that (1) in DPD, the resting-state BOLD complexity is significantly diminished, particularly in several regions pertaining to the regulation of mood; (2) and the depression-related loss of BOLD complexity within those regions is cross-sectionally associated with the reduction of the functional connections of the region. These thus indicated that the complexity of the spontaneous brain activities may provide unique insight into the regulation of mood in those with PD, and may inform the management and therapeutic strategies for DPD with the focus on the complexity of spontaneous brain activity.

Mounting evidence has shown that the complexity, which characterize the multiscale dynamics in the spontaneous behavior or output (e.g., the BOLD fluctuation here) of a given physiologic system during resting state, rather than the single-scale measures, is associated with the capacity of this system to adapt to stress and thus is related to its functional performance. In neurodegenerative disorders, the quantity and/or quality of the regulatory elements in a physiologic system (e.g., the clusters in the brain) and their structural and functional connections to other systems are often altered [27], which manifests as a loss of the physiologic complexity in the dynamics of the system’s spontaneous behavior (e.g., BOLD fluctuations) [28]. Therefore, the degree of complexity in a given physiologic system is determined by both the inputs from other systems (external factors) and the capacity of the regulatory elements within the region (internal factors) to integrate and process these inputs. We here observed that the degree of complexity in 8 of the 18 identified regions was associated with the level of its global FC, which is consistent with the previous studies [28]. Wang and colleagues, for example, demonstrated that greater complexity of BOLD fluctuations in brain functional networks was correlated with greater functional connectivity within the same network in a relatively healthy cohort [23]. Therefore, the results in this study confirmed the findings in previous studies, that is, the complexity metric captures the capacity of information processing within the brain and lower complexity represents less transition or interaction between brain regions, potentially contributing to the depression in PD. On the other hand, it is interesting that no significant correlation between complexity and global FC was observed in the other 10 regions, suggesting that the degree of complexity in deed depends upon both external factors (i.e., global FC) and the internal factors. It is worthwhile to characterize these internal factors and examine its contribution to the degree of resting-state BOLD complexity in future studies.

In this study, we observed that the resting-state BOLD complexity in right frontal lobe, bilateral SMA, bilateral cingulate regions, right THA, and left temporal lobe is lower in DPD group as compared to NDPD group. These findings were in line with previous studies, which linked the neuro- and bio-physiologic alterations within these regions to depression in both people with and without PD. Meanwhile, multiple neural circuits pertaining to the mood regulation have been proposed in previous studies, such as the frontal-subcortical circuit and basal ganglic-thalamic-cortical circuit [9]. The observations from the view of multiscale dynamics of resting-state BOLD fluctuation here may indicate a neural circuit pertaining to DPD, that is, the SMA-right fronto-limbic circuit. Specifically, the activation in frontal lobe, particularly in the right side, is believed to be associated with the processing of emotional inputs and the responses to such emotional stimulus [24–26], and the altered activation, hypoperfusion, and hypometabolism in this region are linked to depressive symptoms [27, 28], as well as DPD [29, 30]. The thalamus is also critical to the regulation of emotion processing and the cingulate regions (i.e., ACC and MCC) are the bridges for the transmission of the information related to emotion [31]. The decreased activation within thalamus, as well as the reduced structural and functional connections of cingulate regions to thalamus, has been linked to the presence of DPD [12, 32–34]. The STG played an important role in the regulation of socio-emotional function, and the alteration in this region (e.g., reduced FC) is observed in DPD [35, 36]. To date, limited evidence has shown the association between SMA, a region primarily related to motor function, and persistent antidepressant effect of medication [37]. We here thus provide novel evidence suggesting that the alteration of the activities within SMA may contribute to DPD. Taken together, the subtle functional and structural changes within this circuit pertaining to DPD may manifest as the loss of resting-state BOLD complexity, which would serve as a target of future’s therapeutic strategies for DPD.

Regarding to the implementation to improve the functionalities by intervening the physiological complexity, studies have shown that the complexity is not an obligation consequence due to aging or disease, but can be restored and improved using appropriate interventions, and the increase of complexity has been linked improved functional performance. In our previous study, repetitive transcranial magnetic stimulation (rTMS) targeted on the bilateral primary motor cortex and cerebellum could relieve motor symptoms in Parkinsonian syndrome, positively correlated with motor network resting-state complexity, and a trend of MSE increase was observed [38]. Therefore, future studies are warranted to examine if the increase of BOLD complexity within this identified circuit as induced by the intervention is associated with the improvement in depressive symptoms.

Though the participants were of high homogeneity (e.g., naïve to anti-depression medications), the sample size in this pilot study is relatively small and the number of participants was not matched between groups, which may limit the power of statistical analyses (e.g., within each cohort, several significant correlations between complexity and global FC observed in the entire population were no longer there). Meanwhile, the participants were at relatively early stage of PD. Future studies with larger sample size of participants over a boarder range of disease severity are thus needed to confirm the observations in this study and explore the influence of disease severity on the resting-state BOLD complexity.

## Supplementary Information

Below is the link to the electronic supplementary material.

Supplementary file1 (DOCX 39 kb)

## Abbreviations

AAL-90: Labels of the automated anatomical labeling atlas
PreCG: Precentral gyrus
SFG: Superior frontal gyrus, dorsolateral
SFGorb: Superior frontal gyrus, orbital part
MFG: Middle frontal gyrus
MFGorb: Middle frontal gyrus, orbital part
IFGoper: Inferior frontal gyrus, opercular part
IFGtri: Inferior frontal gyrus, triangular part
IFGorb: Inferior frontal gyrus, orbital part.
ROL: Rolandic operculum
SMA: Supplementary motor area
OLF: Olfactory cortex
SFGmed: Superior frontal gyrus, medial
SFGmorb: Superior frontal gyrus, medial orbital
REG: Gyrus rectus
INS: Insula
ACC: Anterior cingulate and paracingulate gyri
MCC: Median cingulate and paracingulate gyri
PCC: Posterior cingulate gyrus
HIP: Hippocampus
PHIP: Parahippocampal gyrus
AMYG: Amygdala
CAL: Calcarine fissure and surrounding cortex
CUN: Cuneus
LING: Lingual gyrus
SOG: Superior occipital gyrus
MOG: Middle occipital gyrus
IOG: Inferior occipital gyrus
FG: Fusiform gyrus
PoCG: Postcentral gyrus
SPG: Superior parietal gyrus
IPG: Inferior parietal, but supramarginal and angular gyri
SMG: Supramarginal gyrus
ANG: Angular gyrus
PCUN: Precuneus
PCL: Paracentral lobule
CAU: Caudate nucleus
PUT: Lenticular nucleus, putamen
PAL: Lenticular nucleus, pallidum
THA: Thalamus
HES: Heschl gyrus
STG: Superior temporal gyrus
STGp: Temporal pole, superior temporal gyrus
MTG: Middle temporal gyrus
MTGp: Temporal pole: middle temporal gyrus
ITG: Inferior temporal gyrus
