# Supplementary material for: The altered multiscale dynamics of spontaneous brain activity in depression with Parkinson’s disease
Source: Neurol Sci. 2022 Mar 2;43(7):4211–9. doi: 10.1007/s10072-022-05974-4 (PMC9213374; doi:10.1007/s10072-022-05974-4)
Supplement: Supplementary file 1 — Supplementary file1 (DOCX 39 kb) [file 10072_2022_5974_MOESM1_ESM.docx]

**Supplementary materials**

**Multiscale entropy (MSE)**

The specific calculation procedure of MSE was as following. The length of BOLD time series was 200 points, and the first ten points were discarded to eliminate pre-steady state observations. Each pre-processed BOLD time series of 190 sampled points was first “coarse-grained” into five scales12, 13, by averaging values across non-overlapping windows of different length for each time scale. For example, scale 1 was the original time series (190 points), and scale 2 was derived from the average of every 2 points in the original time series (95 points). The sample entropy of each coarse-grained time-series was then obtained. Sample entropy is defined as the negative natural logarithm of the conditional probability that a time-series, having repeated itself within a tolerance r for m points (defined pattern length), will also repeat itself for m + 1 points without self-matches12, 13. We chose m = 1 and r = 0.35 to obtain reliable estimates of entropies following the suggestions in previous studies on the MSE calculation of BOLD fluctuations9,12,13. The complexity index of the BOLD time series of each voxel was then determined by averaging the entropy across five scales. Greater averaged entropies reflected greater complexity.

**Supplementary Table.** The complexity of each brain region as parcellated by AAL template in DPD and NDPD groups.

| **AAL-90** | **ROI** | **NDPD group（N=19）** | **DPD group （N=10）** | **F value** | **P value** | **Effect**  **size** |
| --- | --- | --- | --- | --- | --- | --- |
| 1 | PreCG.L | 1.258±0.051 | 1.216±0.061 | 3.538 | 0.0717 | 0.770 |
| 2 | PreCG.R | 1.256±0.055 | 1.205±0.044 | 7.179 | 0.013 | 0.988 |
| 3 | SFG.L | 1.279±0.045 | 1.218±0.043 | 11.717 | 0.002 | 1.376 |
| 4 | SFG.R | 1.275±0.033 | 1.209±0.043 | 25.801 | 0.000 | 1.801 |
| 5 | SFGorb.L | 1.254±0.046 | 1.241±0.050 | 0.533 | 0.472 | 0.274 |
| 6 | SFGorb.R | 1.275±0.043 | 1.214±0.059 | 10.374 | 0.004 | 1.247 |
| 7 | MFG.L | 1.304±0.035 | 1.232±0.058 | 15.351 | 0.001 | 1.636 |
| 8 | MFG.R | 1.305±0.029 | 1.245±0.012 | 16.995 | 0.000 | 2.432 |
| 9 | MFGorb.L | 1.280±0.048 | 1.237±0.047 | 4.118 | 0.053 | 0.902 |
| 10 | MFGorb.R | 1.302±0.046 | 1.233±0.070 | 10.683 | 0.003 | 1.251 |
| 11 | IFGoper.L | 1.265±0.050 | 1.212±0.056 | 6.102 | 0.021 | 1.018 |
| 12 | IFGoper.R | 1.262±0.044 | 1.201±0.048 | 10.732 | 0.003 | 1.344 |
| 13 | IFGtri.L | 1.273±0.043 | 1.228±0.052 | 4.823 | 0.038 | 0.974 |
| 14 | IFGtri.R | 1.262±0.040 | 1.214±0.048 | 7.661 | 0.011 | 1.121 |
| 15 | IFGorb.L | 1.245±0.045 | 1.188±0.053 | 7.135 | 0.013 | 1.192 |
| 16 | IFGorb.R | 1.253±0.029 | 1.195±0.052 | 13.911 | 0.001 | 1.517 |
| 17 | ROL.L | 1.210±0.053 | 1.164±0.046 | 4.672 | 0.040 | 0.906 |
| 18 | ROL.R | 1.218±0.044 | 1.168±0.041 | 8.811 | 0.007 | 1.162 |
| 19 | SMA.L | 1.272±0.052 | 1.197±0.052 | 15.116 | 0.001 | 1.442 |
| 20 | SMA.R | 1.273±0.047 | 1.184±0.039 | 24.981 | 0.000 | 2.000 |
| 21 | OLF.L | 1.210±0.052 | 1.157±0.058 | 8.471 | 0.008 | 0.980 |
| 22 | OLF.L | 1.202±0.045 | 1.155±0.058 | 7.107 | 0.013 | 0.869 |
| 23 | SFGmed.L | 1.297±0.035 | 1.213±0.063 | 21.090 | 0.000 | 1.816 |
| 24 | SFGmed.R | 1.297±0.030 | 1.218±0.060 | 21.379 | 0.000 | 1.862 |
| 25 | SFGmorb.L | 1.245±0.037 | 1.245±0.054 | 7.725 | 0.010 | 0 |
| 26 | SFGmorb.R | 1.292±0.036 | 1.231±0.048 | 15.627 | 0.001 | 1.510 |
| 27 | REG.L | 1.270±0.033 | 1.243±0.045 | 2.913 | 0.100 | 0.721 |
| 28 | REG.R | 1.262±0.042 | 1.238±0.047 | 1.938 | 0.176 | 0.549 |
| 29 | INS.L | 1.193±0.047 | 1.130±0.051 | 9.852 | 0.004 | 1.302 |
| 30 | INS.R | 1.195±0.047 | 1.129±0.049 | 12.720 | 0.002 | 1.384 |
| 31 | ACC.L | 1.283±0.047 | 1.168±0.064 | 25.667 | 0.000 | 2.159 |
| 32 | ACC.R | 1.278±0.032 | 1.176±0.484 | 41.980 | 0.000 | 0.363 |
| 33 | MCC.L | 1.279±0.045 | 1.183±0.054 | 27.395 | 0.000 | 1.992 |
| 34 | MCC.R | 1.285±0.038 | 1.192±0.055 | 29.572 | 0.000 | 2.095 |
| 35 | PCC.L | 1.310±0.056 | 1.253±0.059 | 8.845 | 0.006 | 1.000 |
| 36 | PCC.R | 1.295±0.052 | 1.235±0.064 | 8.592 | 0.007 | 1.066 |
| 37 | HIP.L | 1.153±0.042 | 1.114±0.027 | 7.374 | 0.012 | 1.035 |
| 38 | HIP.R | 1.149±0.046 | 1.105±0.037 | 6.722 | 0.016 | 1.018 |
| 39 | PHIP.L | 1.175±0.065 | 1.119±0.040 | 6.711 | 0.016 | 0.968 |
| 40 | PHIP.R | 1.112±0.066 | 1.112±0.046 | 9.200 | 0.006 | 0 |
| 41 | AMYG.L | 1.170±0.068 | 1.142±0.051 | 0.651 | 0.427 | 0.446 |
| 42 | AMYG.R | 1.136±0.072 | 1.136±0.047 | 1.735 | 0.200 | 0 |
| 43 | CAL.L | 1.317±0.054 | 1.282±0.058 | 2.177 | 0.153 | 0.632 |
| 44 | CAL.R | 1.306±0.053 | 1.254±0.061 | 4.479 | 0.045 | 0.932 |
| 45 | CUN.L | 1.332±0.036 | 1.313±0.047 | 0.565 | 0.459 | 0.474 |
| 46 | CUN.R | 1.328±0.039 | 1.310±0.057 | 0.374 | 0.546 | 0.393 |
| 47 | LING.L | 1.279±0.057 | 1.234±0.068 | 2.636 | 0.117 | 0.739 |
| 48 | LING.R | 1.280±0.058 | 1.232±0.063 | 3.481 | 0.074 | 0.804 |
| 49 | SOG.L | 1.319±0.045 | 1.297±0.052 | 0.993 | 0.329 | 0.464 |
| 50 | SOG.R | 1.312±0.045 | 1.302±0.065 | 0.132 | 0.720 | 0.190 |
| 51 | MOG.L | 1.304±0.039 | 1.282±0.049 | 1.606 | 0.217 | 0.516 |
| 52 | MOG.R | 1.285±0.043 | 1.285±0.065 | 1.235 | 0.277 | 0 |
| 53 | IOG.L | 1.284±0.055 | 1.271±0.085 | 0.252 | 0.620 | 0.195 |
| 54 | IOG.R | 1.310±0.050 | 1.297±0.078 | 0.190 | 0.667 | 0.214 |
| 55 | FG.L | 1.213±0.041 | 1.174±0.037 | 5.510 | 0.027 | 0.982 |
| 56 | FG.R | 1.225±0.040 | 1.193±0.036 | 3.476 | 0.074 | 0.827 |
| 57 | PoCG.L | 1.260±0.059 | 1.220±0.055 | 3.221 | 0.085 | 0.693 |
| 58 | PoCG.R | 1.262±0.066 | 1.218±0.057 | 3.629 | 0.068 | 0.697 |
| 59 | SPG.L | 1.299±0.049 | 1.266±0.060 | 2.712 | 0.112 | 0.624 |
| 60 | SPG.R | 1.306±0.055 | 1.259±0.041 | 5.201 | 0.031 | 0.926 |
| 61 | IPG.L | 1.313±0.043 | 1.268±0.045 | 6.388 | 0.018 | 1.030 |
| 62 | IPG.R | 1.318±0.043 | 1.269±0.041 | 8.628 | 0.007 | 1.157 |
| 63 | SMG.L | 1.288±0.050 | 1.229±0.052 | 7.788 | 0.010 | 1.164 |
| 64 | SMG.R | 1.289±0.045 | 1.243±0.046 | 5.995 | 0.022 | 1.015 |
| 65 | ANG.L | 1.321±0.046 | 1.293±0.061 | 1.774 | 0.195 | 0.544 |
| 66 | ANG.R | 1.315±0.044 | 1.283±0.055 | 4.252 | 0.050 | 0.667 |
| 67 | PCUN.L | 1.325±0.037 | 1.278±0.042 | 8.736 | 0.007 | 1.213 |
| 68 | PCUN.R | 1.328±0.039 | 1.284±0.054 | 5.764 | 0.024 | 0.987 |
| 69 | PCL.L | 1.277±0.064 | 1.195±0.048 | 12.077 | 0.002 | 1.386 |
| 70 | PCL.R | 1.293±0.057 | 1.227±0.044 | 10.073 | 0.004 | 1.245 |
| 71 | CAU.L | 1.177±0.037 | 1.122±0.042 | 14.052 | 0.0009 | 1.420 |
| 72 | CAU.R | 1.179±0.038 | 1.137±0.044 | 6.799 | 0.015 | 1.047 |
| 73 | PUT.L | 1.199±0.042 | 1.165±0.037 | 4.481 | 0.044 | 0.842 |
| 74 | PUT.R | 1.196±0.041 | 1.173±0.042 | 2.330 | 0.140 | 0.556 |
| 75 | PAL.L | 1.194±0.048 | 1.176±0.048 | 0.463 | 0.503 | 0.375 |
| 76 | PAL.R | 1.195±0.047 | 1.156±0.032 | 5.909 | 0.023 | 0.916 |
| 77 | THA.L | 1.205±0.049 | 1.153±0.036 | 10.977 | 0.003 | 1.153 |
| 78 | THA.R | 1.207±0.038 | 1.152±0.034 | 17.379 | 0.000 | 1.498 |
| 79 | HES.L | 1.201±0.068 | 1.091±0.059 | 20.184 | 0.000 | 1.689 |
| 80 | HES.R | 1.192±0.066 | 1.109±0.079 | 8.191 | 0.008 | 1.176 |
| 81 | STG.L | 1.237±0.046 | 1.154±0.054 | 17.955 | 0.000 | 1.700 |
| 82 | STG.R | 1.242±0.043 | 1.187±0.052 | 9.097 | 0.006 | 1.191 |
| 83 | STGp.L | 1.192±0.060 | 1.095±0.070 | 13.955 | 0.001 | 1.527 |
| 84 | STGp.R | 1.204±0.051 | 1.115±0.069 | 17.408 | 0.000 | 1.544 |
| 85 | MTG.L | 1.262±0.039 | 1.219±0.044 | 6.056 | 0.021 | 1.056 |
| 86 | MTG.R | 1.272±0.037 | 1.241±0.039 | 2.904 | 0.101 | 0.823 |
| 87 | MTGp.L | 1.195±0.062 | 1.133±0.059 | 6.859 | 0.015 | 1.016 |
| 88 | MTGp.R | 1.226±0.049 | 1.157±0.055 | 11.132 | 0.003 | 1.351 |
| 89 | ITG.L | 1.226±0.042 | 1.192±0.037 | 5.086 | 0.033 | 0.842 |
| 90 | ITG.R | 1.247±0.037 | 1.216±0.044 | 3.412 | 0.077 | 0.785 |

AAL-90: labels of the automated anatomical labeling atlas. PreCG: precentral gyrus. SFG: Superior frontal gyrus, dorsolateral. SFGorb: Superior frontal gyrus, orbital part. MFG: middle frontal gyrus. MFGorb: Middle frontal gyrus, orbital part. IFGoper: Inferior frontal gyrus, opercular part. IFGtri: Inferior frontal gyrus, triangular part. IFGorb: Inferior frontal gyrus, orbital part. ROL: Rolandic operculum SMA: Supplementary motor area. OLF: Olfactory cortex. SFGmed: Superior frontal gyrus, medial. SFGmorb: Superior frontal gyrus, medial orbital. REG: Gyrus rectus. INS: Insula. ACC: Anterior cingulate and paracingulate gyri. MCC: Median cingulate and paracingulate gyri. PCC: Posterior cingulate gyrus. HIP: Hippocampus. PHIP: Parahippocampal gyrus. AMYG: Amygdala. CAL: Calcarine fissure and surrounding cortex. CUN: Cuneus. LING: Lingual gyrus. SOG: Superior occipital gyrus. MOG: Middle occipital gyrus. IOG: Inferior occipital gyrus. FG: Fusiform gyrus. PoCG: Postcentral gyrus. SPG: Superior parietal gyrus. IPG: Inferior parietal, but supramarginal and angular gyri. SMG: Supramarginal gyrus. ANG: Angular gyrus. PCUN: Precuneus. PCL: Paracentral lobule. CAU: Caudate nucleus. PUT: Lenticular nucleus, putamen. PAL: Lenticular nucleus, pallidum. THA: Thalamus. HES: Heschl gyrus. STG: Superior temporal gyrus. STGp: Temporal pole, superior temporal gyrus. MTG: Middle temporal gyrus. MTGp: Temporal pole: middle temporal gyrus. ITG: Inferior temporal gyrus. ^#^:*P*<0.05, ^*^:*P*<0.01.
